# Nestedness in Arbuscular Mycorrhizal Fungal Communities along Soil pH Gradients in Early Primary Succession: Acid-Tolerant Fungi Are pH Generalists

**DOI:** 10.1371/journal.pone.0165035

**Published:** 2016-10-18

**Authors:** Ai Kawahara, Gi-Hong An, Sachie Miyakawa, Jun Sonoda, Tatsuhiro Ezawa

**Affiliations:** Graduate School of Agriculture, Hokkaido University, Sapporo, Japan; Leibniz-Institut fur Pflanzengenetik und Kulturpflanzenforschung Gatersleben, GERMANY

## Abstract

Soil acidity is a major constraint on plant productivity. Arbuscular mycorrhizal (AM) fungi support plant colonization in acidic soil, but soil acidity also constrains fungal growth and diversity. Fungi in extreme environments generally evolve towards specialists, suggesting that AM fungi in acidic soil are acidic-soil specialists. In our previous surveys, however, some AM fungi detected in strongly acidic soils could also be detected in a soil with moderate pH, which raised a hypothesis that the fungi in acidic soils are pH generalists. To test the hypothesis, we conducted a pH-manipulation experiment and also analyzed AM fungal distribution along a pH gradient in the field using a synthesized dataset of the previous and recent surveys. Rhizosphere soils of the generalist plant *Miscanthus sinensis* were collected both from a neutral soil and an acidic soil, and *M*. *sinensis* seedlings were grown at three different pH. For the analysis of field communities, rhizosphere soils of *M*. *sinensis* were collected from six field sites across Japan, which covered a soil pH range of 3.0–7.4, and subjected to soil trap culture. AM fungal community compositions were determined based on LSU rDNA sequences. In the pH-manipulation experiment the acidification of medium had a significant impact on the compositions of the community from the neutral soil, but the neutralization of the medium had no effect on those of the community from the acidic soil. Furthermore, the communities in lower -pH soils were subsets of (nested in) those in higher-pH soils. In the field communities a significant nestedness pattern was observed along the pH gradient. These observations suggest that the fungi in strongly acidic soils are pH generalists that occur not only in acidic soil but also in wide ranges of soil pH. Nestedness in AM fungal community along pH gradients may have important implications for plant community resilience and early primary succession after disturbance in acidic soils.

## Introduction

Soil acidity, represented by high-concentrations of proton, aluminum, and manganese in the soil solution, is a strong constraint on diversity of soil bacteria [1,2] and fungi [3,4], although fungi seem to be less sensitive to pH than bacteria [5]. Soil acidity also constrains plant productivity; inhibition of root elongation and reduction of phosphorus (P) solubility in the soil solution lead to serious P deficiency in plants [6,7,8]. Arbuscular mycorrhizal (AM) fungi are the obligate biotrophs that associate with most land plants, deliver phosphorus (P) to the host plant [9], and thus play a significant role in the establishment of early-successional species in acidic soil [10,11].

AM fungal diversity decreases with increasing soil acidity [3], which is due to the variability of acid-tolerance among species/isolates of the fungi [12,13,14,15]. It has also been demonstrated that soil pH acts as a significant driver for AM fungal community not only at the local-scale level [16,17] but also at the landscape level [18]; typically, differences in pH between two soils are positively correlated with dissimilarities in AM fungal community composition between the soils. Information about AM fungal species that inhabit acidic soils, however, is quite limited [19,20,21], and further, distribution of the species along pH gradients (i.e. from acidic to neutral pH) remains unexplored.

Gostinčar *et al*. [22] provided a conceptual framework for the evolution of fungi in extreme environments such as high/low temperatures, high-salt concentrations, and acidic/basic conditions; fungi in extreme environments may evolve towards specialists that tolerate or prefer the environments, but are unable to compete or simply unable to survive in moderate environments. Accordingly, acidic soil is likely to be an extreme environment in which only a limited range of AM fungal species (i.e. acid-tolerant fungi) are able to survive. On the other hand, An *et al*. [23] observed that some of the fungi detected in strongly acidic soils could also be detected in a soil with moderate pH, leading to the idea that acid-tolerant AM fungi are pH generalists that adapt to wide ranges of soil pH. However, the distribution of the generalists fungi along pH gradients, particularly in the range from moderately acidic to neutral pH, has not been fully confirmed, and the question whether the generalist fungi in neutral soils are as acid tolerant as those in acidic soils remains unanswered.

In the present study, we addressed the following two hypotheses. Firstly, in acidic soils acid-tolerant (generalist) fungi predominantly occur, while in neutral soils both acid-tolerant (generalist) and acid-sensitive fungi coexisted, that is, AM fungal communities in lower-pH soils are subsets of (i.e. nested within) those in higher-pH soils. Secondly, the generalist fungi in neutral soils are as acid-tolerant as those in acidic soils. To test these hypotheses, soil pH was manipulated to examine responses of an AM fungal community in a neutral soil by employing an acidic soil community as control. For further validation, distribution of the fungi along a pH gradient in the field was also analyzed using a synthesized dataset of our previous surveys [23,24] and a newly conducted survey, which covered a wide range of soil pH. In these experiment and surveys the single plant species *Miscanthus sinensis* Anderss. was focused to cancel the potential effect of plant species on community composition of the fungi, because the fungi show ecological host specificity/preference, that is, different plant species harbor different AM fungal assemblages (e.g., [25,26]). *M*. *sinensis* is distributed from the subarctic to tropical zones in eastern Eurasia and Pacific Asia [27], a typical early-successional perennial grass not only in acidic soil but also in neutral soil [23] (i.e. generalist plant), and thus an ideal host for this study.

## Materials and Methods

### pH-manipulation experiment

Responses of AM fungal communities to soil acidification/neutralization were examined using the communities originated from acid sulfate soil in Rankoshi site and dune (near-neutral) soil in Ishikari site, Hokkaido Isl. in Japan (S1 Fig and Table 1), in which the community compositions of the fungi had been studied previously [23,24]. The acid sulfate soil in Rankoshi was originated from pyroclastic sediment and exposed by quarrying. The vegetation is quite poor; *M*. *sinensis* is a dominant species, but only patchily distributed. Ishikari site consists of coastal sand dunes in which typical zonal vegetation is developed along the coastal line. The landward slopes of the dunes are largely covered with *M*. *sinensis*, whereas *Elymus mollis* dominates the seaward slopes. AM fungal diversity was lower in Rankoshi site than in Ishikari site, probably due to the extreme soil acidity [23,24], but the other soil chemical properties, available P and total nitrogen and carbon, are similar between the two sites (Table 1). With these reasons, we considered that the fungal communities in these sites were suitable for examining community responses to soil acidification/neutralization.

**Table 1:** Geographic/climatic data of the study sites and chemical properties of rhizosphere soils of *Miscanthus sinensis*.

	Site (collection year)					
Variable	Rankoshi (2005)	Hazu (2005)	Nago (2006/2007)	Atsuma (2006)	Ishikari (2007/2008)	Mukawa (2009)
Latitude/ longitude (n)^a^	42°80′N/ 140°47′E	34°80′N/ 137°15′E	26°62′N/ 127°98′E	42°73′N/ 141°87′E	43°25′N/ 141°35′E	42°85′N/ 142°26′E
Climatic zone (c)	Subarctic	Temperate	Subtropical	Subarctic	Subarctic	Subarctic
Location (c)	Hokkaido Isl.	Honshu Isl.	Okinawa Isl.	Hokkaido Isl.	Hokkaido Isl.	Hokkaido Isl.
Temperature/ precipitation (n)	7.4°C/ 1,542 mm	16.0°C/ 1,093 mm	23.0°C/ 2,325 mm	6.8°C/ 1,213 mm	8.3°C/ 822 mm	7.2°C/ 1,092 mm
Surrounding vegetation (c)	Deciduous broad-leaved trees	Evergreen broad-leaved trees	*Casuarina*-plantation and sugarcane	Deciduous broad-leaved trees	Coastal dune grassland	Deciduous broad-leaved trees
Land use/ habitat type (c)	Abandoned quarry	Abandoned quarry	Surrounding slope of sugarcane field	Primary grassland after clear cut	Natural grassland	Abandoned quarry
Soil type (c)	Acid sulfate soil	Acid sulfate soil	Acid sulfate soil	Sandy loam soil	Dune soil	Ultramafic soil
Soil chemical properties (n)^b^						
pH (H_2_O)	3.5−3.9 (3.0−5.4)	4.0−4.4 (3.7−5.0)	5.5−5.9 (3.5−5.7)	5.5−5.9 (5.4−6.1)	6.5−6.9 (5.9−7.3)	7.0−7.4 (7.1−7.4)
Available P (mg kg^-1^)	12.6 ± 1.5	38.7 ± 4.7	15.6 ± 2.8	6.7 ± 1.5	19.5 ± 1.9	1.7 ± 0.6
Total N (g kg^-1^)	0.24 ± 0.03	0.11 ± 0.01	0.78 ± 0.05	1.34 ± 0.14	0.41 ± 0.04	0.41 ± 0.11
Total C (g kg^-1^)	3.2 ± 0.5	3.1 ± 0.4	8.9 ± 0.8	19.5 ± 1.80	5.5 ± 0.7	8.3 ± 1.6
Data source	An *et al*. (2008)	An *et al*. (2008)	An *et al*. (2008) and this study	An *et al*. (2008)	Kawahara & Ezawa (2013)	This study

^a^Letters in parentheses represent the type of variable employed in community analyses: n, numerical variable; c, categorical variable.

^b^Mean value ± SE is presented, except for pH for which a mode with a range (minimum–maximum) is indicated.

In Rankoshi site *M*. *sinensis* rhizosphere soils (top 10−20 cm and 30−40 cm in diam, 2−3 kg) were collected underneath 12 plants that were patchily distributed within a 50 × 100 m-plot in May 2008. Similarly, *M*. *sinensis* rhizosphere soils were collected from 12 plants grown on the landward slope at 300 m intervals along the coastal line in Ishikari site in June 2008. Sample collection from Rankoshi and Ishikari sites were permitted by the owner of the land and Ishikari City, respectively. Soil pH of the individual samples ranged from 3.5 to 3.9 in Rankoshi and from 6.5 to 6.9 in Ishikari. These samples were air-dried, and sieved on the 4.5-mm mesh. Equal amounts of each of the 12 soil samples were combined and divided into two parts, and one half was autoclaved. The intact Ishikari soil and the autoclaved Rankoshi soil were mixed at a ratio of 1:1 (v:v), which was designated as neutral-soil AM fungal inoculum. Whereas a mixture of the intact Rankoshi soil and the autoclaved Ishikari soil at 1:1 was designated as acidic-soil AM fungal inoculum. Theoretically, these two inocula were the same in terms of elemental composition. Acid sulfate soil (pH 3.2) collected from a spot without vegetation in Rankoshi site was used as a growth medium that has high-buffering capacity for acidic pH. The absence of fungal propagule in the medium and autoclaved inocula was confirmed in preliminary experiments. Each inoculum was mixed with the growth medium at a ratio of 1:2 and divided into three parts, and then calcium carbonate was added at 0, 3, and 12 g kg^-1^ to adjust pH to 3.4, 4.0, and 5.5, respectively. *M*. *sinensis* (Kaiseisha Co., Ltd., Otofuke, Japan) were sown and grown in the presence of either the neutral soil- or acidic soil-AM fungal inoculum in the 9-cm plastic pots (350 ml in vol) at the three different pH (*n* = 5), thinned to 10 plants pot^-1^ two weeks after sowing, and further grown only with tap water in a temperature/light/humidity-controlled glasshouse (26/20°C, day/night temperature; 60% relative humidity; 14-h photoperiod) with steel flooring (no soil on the floor). The plants with different inocula were grown in different rooms in the facility to avoid cross contamination. The roots were harvested two months after sowing from each pot separately, washed with tap water, freeze-dried for 48 h, and stored at −30°C for DNA extraction.

### Surveys by soil trap culture

To validate community nestedness along a soil pH gradient at the field (landscape) level, the datasets of previous studies [23,24] and newly conducted surveys (in this study) were combined and reanalyzed. Six field sites, Rankoshi, Hazu, Nago, Atsuma, Ishikari, and Mukawa, were chosen across Japan, in which *M*. *sinensis* dominates (S1 Fig). Original data of the community compositions revealed by soil trap culture using *M*. *sinensis* rhizosphere soils in Rankoshi, Atsuma, Hazu, and Nago sites were obtained in An *et al*. [23], and those in Ishikari site were obtained in Kawahara and Ezawa [24]. All data in Mukawa site and a part of data in Nago site were newly obtained in this study. Rankoshi (acid sulfate soil), Atsuma (sandy loam soil), Ishikari (sand dune soil), and Mukawa (ultramafic soil) sites are located in Hokkaido Isl. that belongs to the subarctic zone, and Hazu (acid sulfate soil) and Nago (acid sulfate soil) sites are located in Honshu Isl. in the temperate zone and in Okinawa Isl. in the subtropical zone, respectively. Sample collection from Hazu and Nago sites were permitted by Aichi Pref. and Okinawa Pref. Agri. Res. Center, respectively. Whereas no specific permission was required for Atsuma and Mukawa sites, because the former is primary grassland developed on a terrace along a river after clear-cutting in the 1980s, and the latter is an old quarry abandoned in the 1990s. Information about surrounding vegetation and geographic features in these sites (except for Mukawa site) are described in detail in the previous studies [23,24], but also summarized in Table 1. Site information of Mukawa is described in S1 Text. These six sites covered a soil pH range of 3.0–7.4, which also fully covered the pH range in which *M*. *sinensis* occurs in Japan [28].

*M*. *sinensis* generally develops a clump of multiple stems. In Hazu, Nago, and Mukawa sites vegetation is quite poor, and *M*. *sinensis* is only patchily distributed as observed in Rankoshi site; the diameter of the clumps at the ground ranges 5–10 cm. Whereas in Atsuma and Ishikari sites *M*. *sinensis* forms single-species grassland in which the diameter of the clumps ranges 30–80 cm. In Rankoshi, Hazu, Nago, and Mukawa sites 12 plants that were standing alone (no neighboring plants within 1 m) were randomly chosen (at least 5 m intervals) from each of a 50 × 100 m-plot in Rankoshi, a 200 × 100 m-plot in Hazu, and a 10 × 130 m-plot in Nago sites, and rhizosphere soil samples (top 10–20 cm and 30–40 cm in diam, 2–5 kg) were collected underneath the plants in May 2005, June 2005, and April 2006, respectively [23]. Two additional soil samples were collected from Nago site in April 2007 in this study. In Atsuma site soil samples were collected from 12 intersections of 10 × 5 m interval grid lines drawn within a 50 × 30 m-plot in October 2006 [23]. In Ishikari site soil samples were collected from six and 12 plants grown on the landward slope at 600 m intervals in June 2007 and 300 m intervals in June 2008, respectively, along the coastal line [24]. In Mukawa site soil samples were collected from 15 plants along the slope at 20–30 m intervals in June 2009 in this study.

These soil samples were air-dried, sieved on a 4.5-mm stainless steel mesh, and used for soil trap culture within two weeks after collection [24]. Briefly, seeds of *M*. *sinensis* were sown onto each of the soil samples in 9-cm plastic pots and grown only with tap water in the glasshouse. Plants grown in the soils from different sites were grown separately in different rooms in the facility to avoid cross contamination. The roots were harvested two months after sowing from each pot separately, freeze-dried, and stored at −30°C for DNA extraction.

For the analysis of soil chemical properties, subsamples of the rhizosphere soils were air-dried in the glasshouse, crushed, and passed through a 2-mm sieve. Soil pH (H_2_O) was measured at a 1:2.5 soil:water ratio (w/v) using an electrode after shaking for 1 h at 160 rpm. Total carbon (C) and nitrogen (N) were analyzed using Vario MAX CNS analyzer (Elementar, Tokyo). Available P was extracted either with 1 mM sulfuric acid at a 1:200 (w/v) ratio of soil to extraction buffer (modified from Truog [29]) or with 0.03 M ammonium fluoride/ 0.1 M hydrochloric acid solution at a 1:7 (w/v) ratio of soil to extraction buffer [30] and measured colorimetrically; the former buffer is suitable for acidic and weakly acidic soils (Rankoshi, Hazu, Nago, Atsuma, and Ishikari sites), whereas the latter buffer is for neutral and alkaline soils (Mukawa site).

### LSU rDNA sequencing and phylotype definition

DNA extraction from the root samples, amplification of a partial sequence of fungal large subunit ribosomal RNA gene (LSU rDNA), cloning, and sequencing were carried out according to Kawahara and Ezawa [24]. Briefly, the freeze-dried root samples were ground using Multi-Beads Shocker (Yasui Kikai) with a metal cone, and DNA was extracted with DNeasy Plant Mini Kit (Qiagen, Tokyo). LSU rDNA was amplified using Expand High-Fidelity PLUS PCR System (Roche Diagnostics, Tokyo) with the forward LR1 [31] and reverse FLR2 [32] primers. Each PCR product was purified with MonoFas DNA purification Kit (GL Sciences, Tokyo) and cloned into pT7Blue T-vector (Novagen/Merch, Tokyo) to construct a library, and nucleotide sequences of randomly chosen clones were determined using BigDye Terminator v3.1 Cycle Sequencing Kit with ABI PRISM 3130*xl* Genetic Analyzer (Applied Biosystems, Tokyo).

All sequences obtained from the pH-manipulation experiment and the trap culture surveys were first aligned together with the published AM fungal sequences selected across all families (reference sequences) using Clustal X ver. 1.81 [33] to construct a preliminary neighbor-joining tree. At this step, the sequences that clustered outside glomeromycotan sequences were excluded, and those that were likely to belong to Glomeromycota were grouped based on a criterion of sequence similarities at ≥ 95% [34,35] using Sequencher v. 5.0 software (Gene Codes Corporation, Ann Arbor, MI). Representative sequences that were randomly chosen from each of the 95%-similarity-groups and those that did not form a group i.e. singletons were subjected to BLAST searches, and validity of the sequences (groups), e.g., whether they were chimeric or not, was carefully assessed by comparing published sequences. Singletons that showed similarity to published sequences at < 95% were excluded in this step. To construct the neighbor-joining tree for phylogenetic analysis, the representative sequences were aligned together with the reference sequences using Clustal X, and confidence limits of each branch in the phylogeny were assessed by 1000 bootstrap replications and expressed as percentage values. The tree was displayed using NJplot software [36]. The tree topologies were generally in good agreement with the 95%-similarity-groups with respect to the bootstrap values (> 70%), and thus each group was defined as a single phylotype.

Rarefaction curves were constructed based on sequenced clone numbers using Analytic Rarefaction 1.3 (http://www.uga.edu/strata/software/index.html). In the pH-manipulation experiment, numbers of clones for sequencing were increased until 95% confidence intervals of rarefaction analysis reached within ± 0.4 phylotype for each treatment. In the trap culture surveys, 12 root samples from each site were initially subjected to DNA extraction, and clone libraries were raised separately from the individual samples in which PCR products were obtained. Numbers of sequences were increased until the confidence intervals reached within ± 0.5 phylotype for each library. For the sites in which the confidence intervals of total richness exceeded ± 0.4 phylotype, additional samples were collected from the field and subjected to trap culture for further sequencing. The representative sequences that were newly obtained in this study are deposited in DDBJ under the accession numbers AB610832–AB610837, AB710218–AB710223, AB750739–AB750746, and AB840271.

### Statistical analysis

Permutational multivariate analysis of variance (PERMANOVA) and Mantel test were performed with PAST v2.17b [37]. In PERMANOVA the presence/absence dataset was used, in which Jaccard distance was employed as a measure of community dissimilarity between the samples (9999 permutations). In Mantel test a phylotype-abundance (frequency) dataset was constructed by pooling the presence/absence data of individual samples within each site, and Morisita-Horn distance was employed as a measure of community dissimilarity. In this test average values of the soil chemical data were calculated for each site as representative data of the sites. Forward selection procedure by means of Monte Carlo permutation test in canonical correspondence analysis (CCA) was performed using the presence/absence dataset with CANOCO 4.5 (Microcomputer Power, Ithaca, NY) (999 permutations). Linear correlation analysis was performed with StatView (SAS institute Japan, Tokyo). In all statistical analysis soil pH values were treated as a real number, although pH is a logarithmic value (−log [H^+^]).

In nestedness analysis, i) community nestedness (i.e. species-poor communities are subsets of species-rich communities) and ii) consistency of species abundance across environmental gradients were tested by using nested overlap and decreasing fill (NODF) [38] and weighted NODF (WNODF) [39], respectively, as indices. The phylotype-presence/absence data of individual samples were first pooled within each pH treatment (pH-manipulation experiment) or within each site (trap culture surveys), which were designated as abundance data. In the trap culture surveys, the abundance data were normalized by dividing by total sample number in each site and expressed as percentage relative abundance, because sample numbers were different among the sites. The matrices constructed with the abundance data were used for WNODF, and those constructed after converting to a presence/absence format were used for NODF. Orders of the columns in these matrices were sorted according to specific hypotheses, and that of the rows was sorted by occurrence of the phylotypes (total number of samples in which each phylotype occurred). NODF among columns (NODF_column_) was calculated to examine whether species-poor communities are subsets of species-rich communities, whereas WNODF among rows (WNODF_row_) was for examining consistency of species (phylotype) abundance across the sites/treatments. The significance of NODF among columns (NODF_column_) and WNODF among rows (WNODF_row_) was tested against 1000 randomly generated matrices. The indices range from 0 (non-nested) to 100 (fully nested) and were deemed significant if the value was higher than the expected values that were obtained from 1000 randomly generated matrices (Monte Carlo procedure) under the column totals fixed and row totals fixed constraint null model [40]. Z-scores of NODF_column_ and WNODF_row_ were calculated as (NODF_column_ − NODF_column -exp_)/ SD_exp_ for NODF_column_ and (WNODF_row_ − WNODF_row-exp_)/ SD_exp_ for WNODF_row_, where SD_exp_ is standard deviation of the expected values. A significant positive Z-score indicates that there is a nested pattern in the matrix, while a significant negative Z-score indicates 'anti-nestedness' [41].

If an anti-nestedness pattern was observed, S-D-R (similarity-difference-replacement) simplex analysis was performed for further exploration of the pattern with SDR-abunSimplex program [42]. Three complementary indices that measure species/abundance similarity (*S*), richness/abundance difference (*D*), and species/abundance replacement (*R*) were first calculated for all pairs of the sites in the matrices, and then turnover (*1−S*), agreement (*1−D*), and nestedness (*1−R*) were also calculated as corresponding indices. For the presence/absence data, *S*, *D*, *R* were calculated according to Podani & Schmera [43]. *S* is Jaccard index: *S = a/n*, where *a* is the number of species shared by two sites, and *n* is total number of species. *D* is a ratio of absolute difference in the number of unique species between two sites: *D = |b − c|/n*, where *b* and *c* is the number of unique species in sites B and C, respectively. *R* is given by the following equation: R = 2*min {b, *c}/n*. For abundance data, *S* represents Ruzicka index, and the equations of *D* and *R* were developed based on the index, which are conceptually corresponding to those for presence/absence data [42]. In this study, contributions of phylotype (abundance) turnover (*1−S*) and nestedness (*1−R*) to the distribution patterns of AM fungal phylotypes were presented as percentage value. It should be noted that the nestedness index in S-D-R simplex analysis is calculated using the equation, different from NODF/WNODF, and thus cannot be directly compared with NODF/WNODF.

## Results

### Arbuscular mycorrhizal fungal phylotypes

Fungal LSU rDNA was successfully amplified by one-step PCR from all 30 samples of the pH-manipulation experiment (two inocula × three pH levels, *n* = 5). In the trap culture surveys amplicons were also obtained from eight, nine, nine, and ten samples from Rankoshi, Hazu, Nago (2006 collection), and Atsuma sites, respectively [23], from four and six samples collected in 2007 and 2008, respectively, from Ishikari site [24], from two additional samples of Nago site collected in 2007, and from 13 samples of Mukawa site. Clone libraries were raised from each amplicon, and about 1000, 500, 400, and 800 AM fungal sequences were obtained in the pH-manipulation experiment, An *et al*. [23], Kawahara and Ezawa [24], and the recent surveys, respectively (2700 sequences in total). By analyzing these sequences, 52 phylotypes were defined and assigned to the Glomeromycota. Among them, 38 phylotypes were assigned to ten known genera across seven families: 15 phylotypes in *Rhizophagus* (Glomeraceae), five phylotypes in *Glomus* (Glo1–5) (Glomeraceae), two phylotypes in *Funneliformis* (Fun1–2) (Glomeraceae), three phylotypes in *Acaulospora* (Aca1–3) (Acaulosporaceae), two phylotypes in *Diversispora* (Div1–2) (Diversisporaceae), one phylotype in *Gigaspora* (Gig1) (Gigasporaceae), three phylotypes in *Scutellospora* (Scu1–3) (Gigasporaceae), two phylotypes in *Claroideoglomus* (Cla1–2) (Claroideoglomeraceae), one phylotype in *Ambispora* (Amb1) (Ambisporaceae), and four phylotypes in *Paraglomus* (Par1–4) (Paraglomeraceae) (S2 Fig). Ten phylotypes that are likely to belong to Glomeraceae, but could not be assigned to the known genera were named as unassigned Glomeraceae (UnG1–10), while four phylotypes that were likely to belong to Glomeromycota but could not be assigned to any of the families were named as uncultured Glomeromycota (Unc1–4). The numbers of sequenced clone and frequencies of the individual phylotypes in the pH-manipulation experiment and the trap culture surveys are presented in S1 and S2 Tables, respectively.

Rarefaction analysis on phylotype richness was conducted; 95% confidence intervals of total richness were ± 0.15 and 0.13 in the neutral soil- and acidic soil-communities, respectively, in the pH-manipulation experiment, and ± 0.18, 0.23, 0.30, 0.29, 0.20, and 0.20 phylotype in Rankoshi, Hazu, Nago, Atsuma, Ishikari and Mukawa sites, respectively, in the surveys (S3 Fig). These results suggested that our sampling provided reasonable coverages of AM fungal diversity.

### pH-manipulation experiment

**Fig 1:**
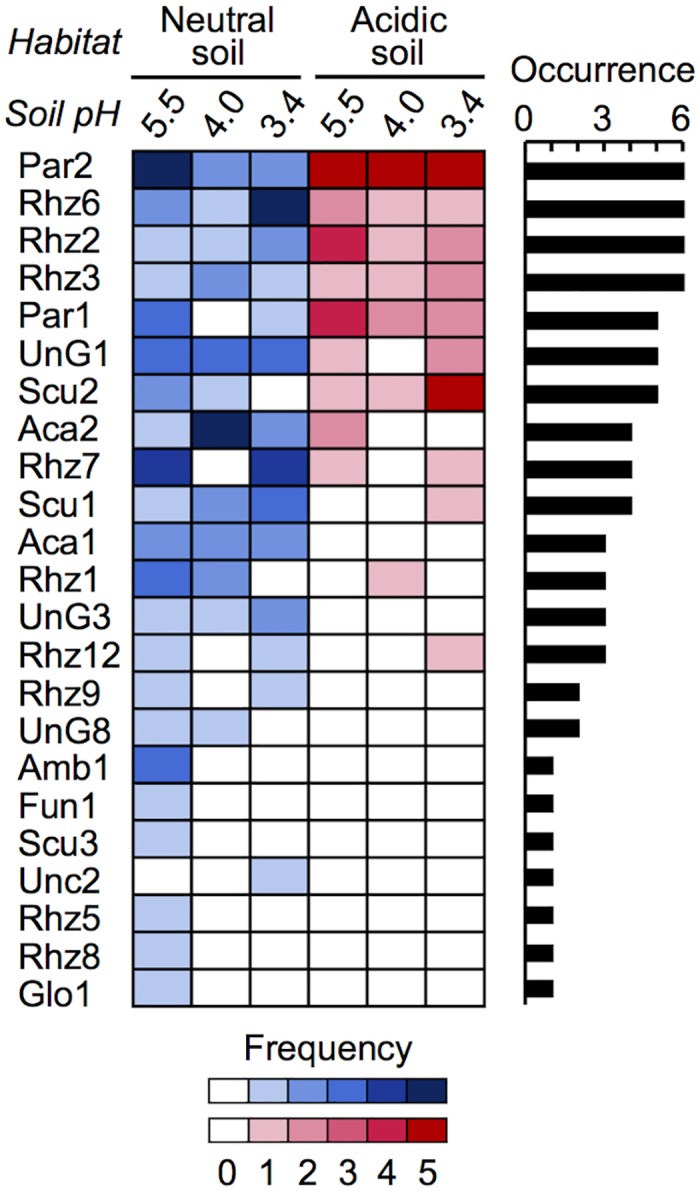
Nestedness in arbuscular mycorrhizal fungal communities as structured by pH manipulation. Rhizosphere soils of *M*. *sinensis* were collected from Ishikari (neutral soil) and Rankoshi (acidic soil) sites and subjected to trap culture at pH 3.4, 4.0, and 5.5 with *M*. *sinensis* seedlings for community analysis (*n* = 5). The names of phylotypes were defined based on the genus to which they were assigned (S2 Fig) and indicated on the left of matrix. A combined matrix of the neutral- and acidic-soil communities was constructed according to the hypothesis; the columns of the neutral- and acidic-soil communities were placed to the left and right, respectively, and sorted by pH, and the rows were sorted by occurrence (number of sample in which they occurred was indicated on the right of the matrix). Darkness of the cells indicates the frequency of the phylotypes (numbers of sample in which they occurred). Significant nestedness was observed among the columns (NODF_column_ = 70.83, *Z* = 2.71, *P* < 0.001), but an anti-nestedness pattern was also observed among the rows (WNODF_row_ = 39.03, *Z* = −4.35, *P* < 0.001).

Two-way PERMANOVA indicated that the pH manipulation (3.4, 4.0, and 5.5) and the difference in inoculum, as well as their interaction, had significant impacts on the community compositions (Table 2). Subsequent one-way PERMANOVA indicated that the pH manipulation had a significant impact on the communities originated from the neutral soil (neutral-soil communities) (Pseudo-*F* = 2.16, *P* = 0.004), but not on the communities from the acidic soil (acidic-soil communities) (Pseudo-*F* = 2.00, *P* > 0.05). For nestedness analysis, a combined matrix of the neutral- and acidic-soil communities was constructed according to the hypothesis that the neutral-soil communities grown at higher pH nest those grown at lower pH and all acidic-soil communities; the columns of the neutral- and acidic-soil communities were placed to the left and right, respectively, and then sorted by pH (Fig 1). In the presence/absence matrix significant nestedness was observed among the columns (inocula/pH levels) (NODF_column_ = 70.83, *Z* = 2.71, *P* < 0.001), supporting the hypothesis. In the abundance-based matrix, however, an anti-nestedness pattern was observed among the rows (phylotypes) (WNODF_row_ = 39.03, *Z* = −4.35, *P* < 0.001), which implies that phylotype abundance is inconsistent across the soils/pH gradients. Subsequent S-D-R simplex analysis indicated that the relative contribution of nestedness was decreased in the abundance-based matrix (65.4%), compared with that in the presence/absence matrix (84.3%) (S3 Table). Instead, relative contribution of phylotype (abundance) turnover was increased in the abundance-based matrix (65.0%), compared with that in the presence/absence matrix (49.5%). These results imply that there is a nested structure in the matrix, but dominant phylotypes are differentiated between the inocula and along the pH gradients.

**Table 2:** Responses of AM fungal communities originated from acidic and neutral soils to pH manipulation.

Factor	df	Pseudo-*F* (*n* = 5)	*P*
pH	2	1.76	0.032
Inoculum	1	5.70	< 0.001
pH × inoculum	2	2.43	0.002

Two-way PERMANOVA on the phylotype-presence/absence data (9999 permutations).

### Trap culture surveys: community nestedness

Prior to nestedness analysis, validity of the combined dataset for this analysis was evaluated. Firstly, the influence of differences in sample collection year on the community compositions in Nago and Ishikari sites was examined by PERMANOVA; no significant differences in the composition were observed among the samples collected in 2006 and 2007 in Nago site (Pseudo-*F* = 0.700, *P* = 0.574) and among those collected in 2007 and 2008 in Ishikari site (Pseudo-*F* = 1.14, *P* = 0.302), implying that annual variations in these sites were minimum. Secondly, the significance of pH in driving the communities was assessed based both on the individual sample-based analysis (CCA) and on the pooled-sample (site)-based analysis (Mantel test). CCA forward selection procedure showed that soil pH was the most significant factor (Pseudo-*F* = 6.21, *P* = 0.002), followed by soil type and climatic parameters (S4 Fig and S4 Table). Mantel test revealed that pH was the only significant factor that correlated with community dissimilarities across the sites (*r* = 0.755, *P* = 0.01), and none of the other variables could explain the residual variation that could not be explained by pH (S5 Table). These results imply that soil pH is the primary driver of the communities across the sites at least within the dataset.

**Fig 2:**
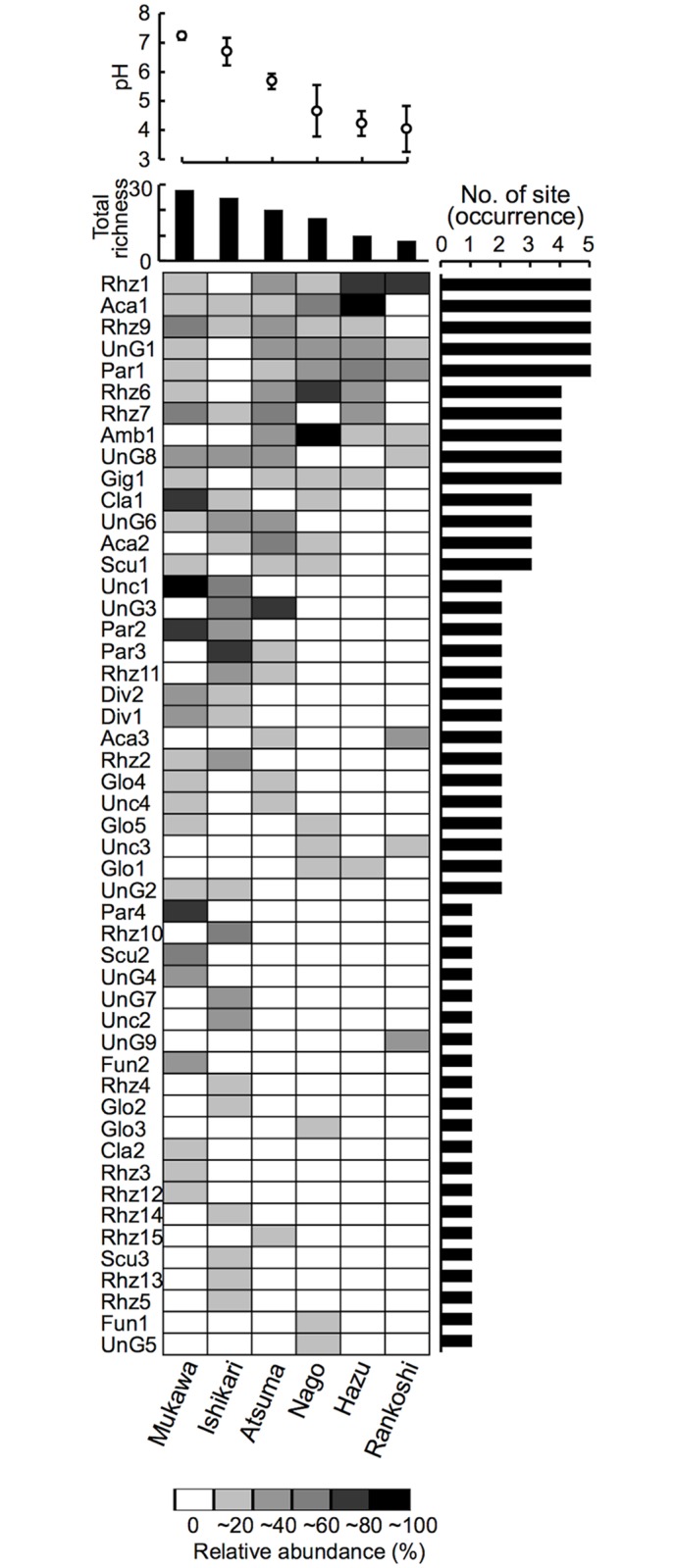
Nestedness in arbuscular mycorrhizal fungal communities along the soil pH gradient in the trap culture surveys. Rhizosphere soils of *M*. *sinensis* were collected from Rankoshi, Hazu, Nago, Atsuma, Ishikari, Mukawa sites and subjected to trap culture with *M*. *sinensis* seedlings for community analysis. The names of phylotypes were defined based on the genus to which they were assigned (S2 Fig) and indicated on the left of matrix. The columns (sites) and rows (phylotypes) were sorted by the mean-pH value of the soil samples (mean ± SD was indicated above the matrix) and by occurrence (number of site at which they occurred was indicated on the right of the matrix), respectively. Darkness of the cells indicates percentage relative abundance of the phylotypes. Significant nestedness was observed among the columns (NODF_column_ = 55.95, *Z* = 1.79, *P* = 0.037), but an anti-nestedness pattern was also observed among the rows (WNODF_row_ = 18.97, *Z* = −16.03, *P* < 0.001).

Based on the result that the community differentiation among the sites was primarily driven by pH, order of the columns (sites) in the matrices for nestedness analysis was sorted by the mean pH value of each site (Fig 2). In the presence/absence matrix, a significant nestedness pattern was observed among the columns (sites) (NODF_column_ = 55.95, *Z* = 1.79, *P* = 0.037), implying that the community nestedness is structured along the pH gradient. If phylotype abundance was taken into account, however, a significant 'anti-nestedness' pattern was observed among the rows (phylotypes) (WNODF_row_ = 18.97, *Z* = −16.03, *P* < 0.001). S-D-R simplex analysis indicated that the relative contribution of nestedness was 61.6% in the presence/absence matrix, but was decreased to 45.8% in the abundance-based matrix (S6 Table). Whereas phylotype (abundance) turnover became a more important component in the abundance-based matrix (85.1%) than in the presence/absence matrix (73.8%).

### Trap culture surveys: generalist phylotypes

**Fig 3:**
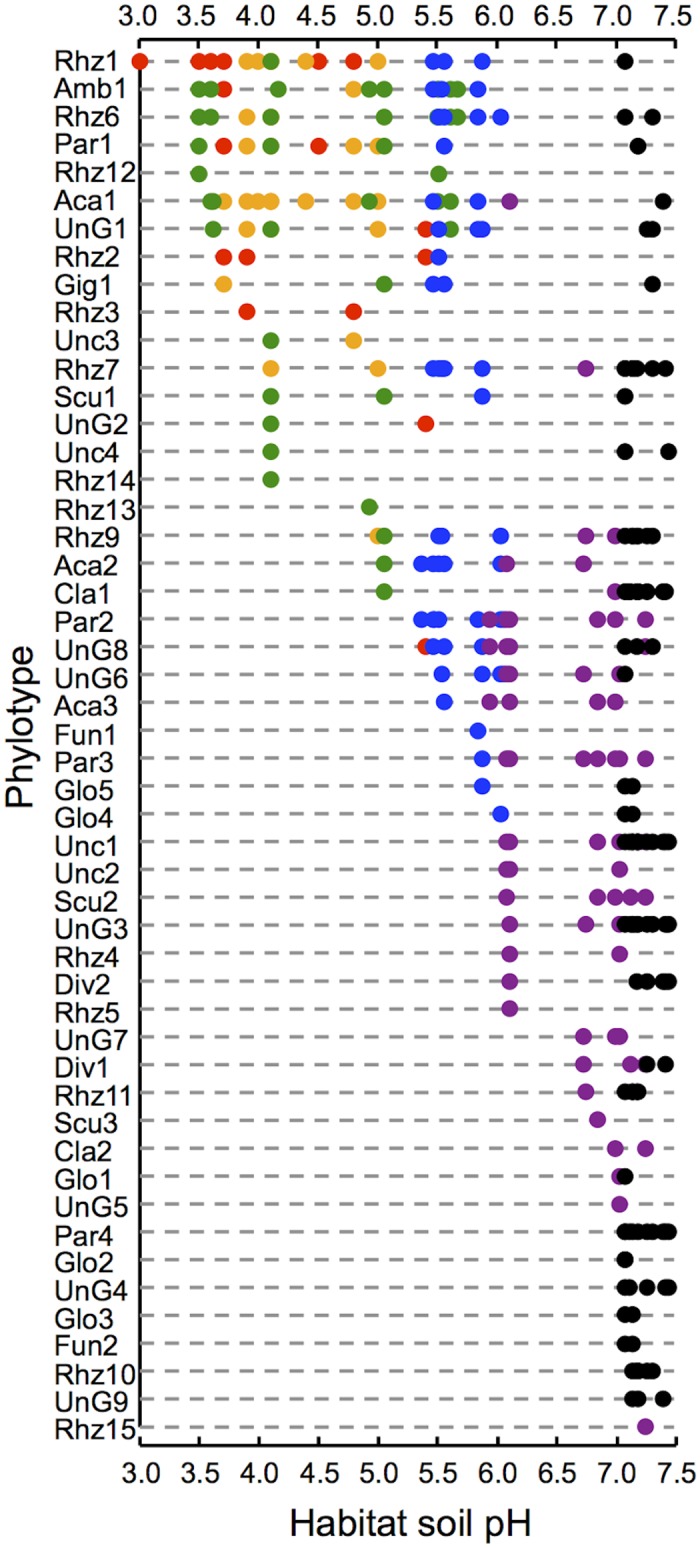
Scatter plot of soil sample pH at which arbuscular mycorrhizal fungal phylotypes were detected in the trap culture surveys. Rhizosphere soils of *M*. *sinensis* were collected from Rankoshi (red), Hazu (yellow), Nago (green), Atsuma (blue), Ishikari (purple), and Mukawa (black) sites and subjected to soil trap culture with *M*. *sinensis* seedlings for community analysis. pH was measured using subsamples of the soils. The phylotypes were sorted by the lowest pH values at which they occurred. The names of phylotypes were defined based on the genus to which they were assigned (S2 Fig).

**Fig 4:**
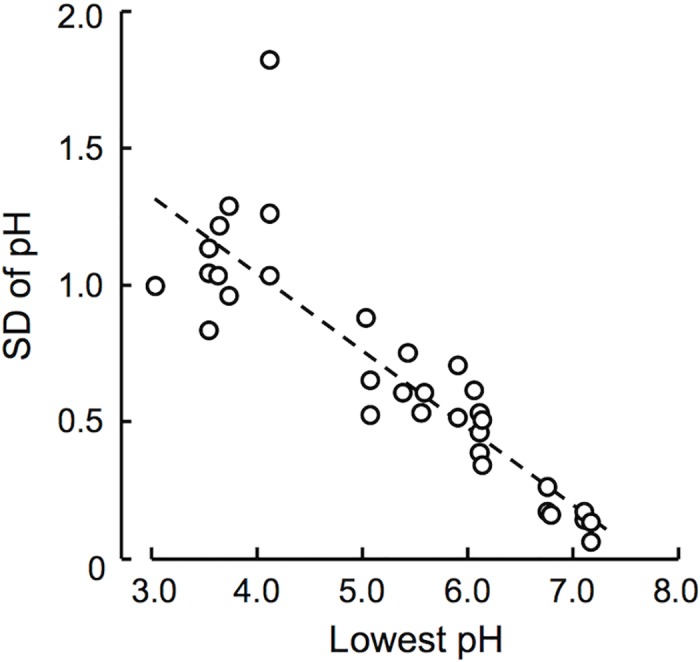
Correlation of acid-tolerance with pH adaptability of arbuscular mycorrhizal fungal phylotypes. Standard deviations (SDs) of soil sample pH (range of soil pH) were plotted against the lowest soil pH (acid-tolerance) for each phylotype that occurred in three or more samples in the trap culture surveys. Correlation coefficient (*r*) = −0.800, *P* < 0.001 (*n* = 33).

For characterization of generalist phylotypes, distribution of the individual phylotypes along the pH gradient was overviewed via plotting pH of the soil samples in which they occurred, and then the phylotypes were sorted by the lowest pH values at which they occurred (Fig 3). The plot suggested that there is a continuum of the level of acid-tolerance, along which pH adaptabilities (generality) of the phylotypes are defined. To validate this idea, standard deviations (SDs) of soil pH were calculated for each phylotype that occurred in at least three soil samples and plotted against the lowest pH values (Fig 4). The SD values were negatively correlated with the lowest pH values (*r* = −0.800, *P* < 0.001), confirming that more acid-tolerant phylotypes occur over wider ranges of pH, that is, they are generalist phylotypes.

## Discussion

The present study demonstrated community nestedness of AM fungi along pH gradients, with particular emphasis on strongly acidic soil, by the experimental approach in conjunction with the trap culture surveys. The fungi in acidic soils occur over wide ranges of pH, namely pH generalists, and the generalist fungi in neutral soils are as acid-tolerant as those in acidic soils.

The nested structure in mutualistic networks, such as plant-animal pollinator networks, has been implicated not only in coexistence and stability of species [44], but also in community stability [45]. These implications could also be applied to plant-AM fungal networks in which highly nested structures have been observed [46,47]. On the other hand, community nestedness along environmental gradients, in which species tolerating harsh environments are a subset of those that occur in moderate environments [48], is likely to be more directly relevant to community stability; the tolerant species survive after environmental perturbations and thus can become early-successional species that play a key role in the re-establishment of community [49]. In fact, a recent study indicated that an AM fungal taxon pool establishes earlier than that of plant and thus is a likely driver of plant successional dynamics [50]. Given the significance of AM fungi in early primary succession in acidic soils [10,23], the community nestedness along pH gradients in the fungi may have important ecological implications not only for AM fungal communities but also for plant community resilience/establishment in acidic soil; acid-tolerant (early-successional) AM fungi are widely distributed across ecosystems and thus can readily support plant colonization in acidic soil, which would consequently make a significant contribution to rapid recovery of vegetation and succession in acidic soils.

One driver for community nestedness is environmental filtering [48,51,52]. Therefore, the community nestedness observed along the pH gradient could be simply interpreted by coexistence of acid-tolerant and -sensitive fungi at moderate pH and selection of acid-tolerant fungi in the acidic soils by pH filtering. This broad distribution of acid-tolerant AM fungi is probably achieved by minimizing costs for expression of acid-tolerant traits in moderate-pH soils, which would be essential to maintain competitiveness over a wide range of pH. In this context, the generalist fungi would be the 'phenotypically plastic genotypes' that can regulate expression of genes responsible for stress tolerance flexibly in response to environmental perturbations [53], probably through rapid changes in the frequency of nucleotypes as observed in *Rhizophagus irregularis* [54] that is a close relative of the generalist phylotype Rhz7 (occurred at pH 4–7.4, Fig 3).

Despite the broad distribution of the generalist fungi, the S-D-R simplex analysis indicated that they are not always dominants, particularly in the moderate-pH soils. One interpretation of these results is that the costs for acid-tolerant traits of the generalists, even if they could minimize, would become significant in less acidic soils, and thus local specialists are more competitive in the soils. Physiological mechanisms underlying acid-tolerance have not yet been elucidated in AM fungi. In the model fungus *Saccharomyces cerevisiae*, maintenance of magnesium homeostasis [55], protection against oxidative stress [56,57], and sequestration of excessive cytosolic H^+^ into vacuoles [58] have been proposed for the mechanisms of acid tolerance and could also be involved in AM fungal acid tolerance. The sequestration of Al^3+^ into vacuoles as an Al-polyphosphate complex is likely to be a common mechanism for detoxification of Al^3+^ in soil fungi [59]. Rapid hyphal growth and turnover may be involved in the avoidance of Al^3+^ toxicity; fungi that are capable of synthesizing and turning over (i.e. recycling) cell wall/plasma membrane more rapidly could repair hyphal damages by Al^3+^ toxicity more rapidly and thus can survive in acidic soils. All these mechanisms are primarily energy-consuming processes and thus likely to have a negative impact on competitiveness of acid-tolerant (generalist) fungi grown in moderate-pH soils.

One might assume that the pH generalists consist of several phylotypes that differ in pH sensitivity, that is, there is the possibility that they are assemblages of specialists that differ in pH preference, but could not be separated by our criteria (≥ 95%-sequence-similarity). To examine this possibility, we analyzed whether there are sequence groups associated with particular pH ranges within the generalist phylotypes (those that occurred at pH less than 4.5) by using all sequence data obtained both in the pH-manipulation experiment and trap culture surveys; no generalist phylotype was separated into several clusters that showed different pH preferences (data not shown), confirming that they are not assemblages of specialists. Another possibility is that they consist of several genotypes (or nucleotypes) that share the same LSU rDNA sequences, but differ in acid tolerance. If this is the case, selection of progenies that exhibit different sensitivity to soil acidity would theoretically be possible, although establishment of genotypic markers, such as amplified fragment length polymorphism (AFLP) markers [54], would be necessary to distinguish them.

The present study employed the trap culture approach both for the pH-manipulation experiment and for the field surveys. In the field, it has been demonstrated that various biotic and abiotic factors, e.g., plant species [25,26], plant growth stage [60], and climatic variables [61], affect the community composition of the fungi. But in trap culture at least the plant factors and culture conditions are consistent across samples, which is a great advantage for studies on community ecology of the fungi. The community compositions revealed by trap culture, however, are usually different from those revealed by direct analysis of field-collected roots. During the process of soil collection for trap culture, hyphal networks of the fungi in the soil are destructed, which would reduce competitiveness of the field dominants and allow the rapid colonizers to dominate the niche, namely cultivation bias [62]. According to the C-S-R framework applied to AM fungal life-history strategies [63], the fungi could be grouped based on the following functional traits; 'competitors (C)' that proliferate mainly via hyphal networks during the growing season and produce spores lately, 'ruderals (R)' that colonize plants rapidly after disturbance and produce many spores earlier, and 'stress-tolerators (S)' that tolerate abiotic stresses but grow slowly. Based on this concept, R fungi may preferentially be detected in the trap culture approach, while C fungi would be underestimated. On the contrary, community composition in the roots at a given time could properly be demonstrated in the direct analysis, but potential of R fungi in the soil is likely to be underestimated. Given that the present study focused on the communities in early primary succession in acidic soils, R fungi and/or R-S fungi, that is, R fungi with acid-tolerant (S) traits, may intrinsically predominate and play a major role in the establishment of vegetation in the sampling sites. Therefore, trap culture is a valuable approach for understanding the distribution and dynamics of AM fungi in early primary succession.

## Conclusion

The present study provides new insights of the adaptive evolution of AM fungi. The physiological/genetic mechanisms underlying the greater adaptability of pH-generalist fungi are of interest. In yeast cell a large set of genes similarly responds to different stresses: temperature, osmotic, and oxidative stresses [64,65], suggesting that fungal genomes possess a set of core stress response genes that confer a basic level of protection. These observations lead to the expectation that AM fungi that are tolerant to one stress are able to tolerate multiple stresses. In this context, pH-generalist fungi are potentially applicable not only to revegetation [23] and agriculture [66] in acidic soil but also to those in other stress environments, e.g., heavy metal-contaminated soil and saline soils. Further studies on the distribution of the generalists in various harsh environments, as well as physiological and genetic characterizations of the generalists, are necessary.

In the present study, only the fungal communities associated with the generalist plant *M*. *sinensis* were investigated to exclude the potential effect of partner selection/preference on the community compositions. This raises, however, a new question whether community nestedness could also be observed in those associated with specialist plants, because our finding does not necessarily imply that specialist fungi are absent in acidic soils. For example, in forest ecosystem it has been found that forest-specialist plants are likely to associate with both forest-specialist and habitat-generalist AM fungi, which is in contrast to the habitat-generalist plants that preferentially associate with the generalist fungi [46,67]. Accordingly, the presence of acidic-soil specialist fungi cannot be excluded, and thus further investigation of the communities associated with a broader range of plant in acidic soil is of interest.

## Supporting Information

S1 Fig Location of the sampling sites from which rhizosphere soils of *Miscanthus sinensis* were collected. Soil types are acid sulfate soil in Rankoshi, Hazu, and Naog sites, dune soil in Ishikari, sandy loam soil in Atsuma, and ultramafic soil in Mukawa. Geographic/climatic data, vegetation, and soil chemical properties are summarized in Table 1. Reprinted from Sekai Chizu (http://www.sekaichizu.jp/) with permission from Itsuki Shoji Co., Ltd., original copyright 2009.
(TIFF)

S2 Fig Phylogenetic positions of arbuscular mycorrhizal fungal phylotypes defined in this study. Representative sequences of large subunit ribosomal RNA gene of arbuscular mycorrhizal (AM) fungi obtained in the pH-manipulation experiment and trap culture surveys were aligned together with published sequences using Clustal X, and the tree was drawn by NJplot. Grey boxes represent AM fungal phylotypes that were defined based on ≥ 95% sequence similarities. Bootstrap values more than 70% are indicated. Genbank accession numbers of the reference and representative sequences are indicated.
(TIFF)

S3 Fig Rarefaction analysis on arbuscular mycorrhizal fungal phylotype richness based on sequenced clone number. a) Trap culture surveys. Rhizosphere soils of *M*. *sinensis* were collected from Rankoshi (red), Hazu (yellow), Nago (green), Atsuma (blue), Ishikari (purple), and Mukawa (black) sites and subjected to trap culture with *M*. *sinensis* seedlings for community analysis. b) pH-manipulation experiment. Rhizosphere soils of *M*. *sinensis* were collected from Ishikari (open squares) and Rankoshi (closed squares) sites, designated as acidic soil- and neutral soil-AM fungal inocula, respectively, and subjected to trap culture with *M*. *sinensis* seedlings at three different pH. Total richness in the combined communities of all pH treatments was analyzed. The curves were constructed with Analytic Rarefaction 1.3. Bars indicate 95% CI.
(TIFF)

S4 Fig Biplot of canonical correspondence analysis on arbuscular mycorrhizal fungal communities in the trap culture surveys. Rhizosphere soils of *M*. *sinensis* were collected from the six sites and subjected to soil trap culture with *M*. *sinensis* seedlings for community analysis. All the environmental variables used in this analysis are indicated as arrows, and the significance of the factors was assessed by forward selection procedure by means of Monte Carlo permutation test (S4 Table).
(TIFF)

S1 Table Frequency of the occurrence of arbuscular mycorrhizal fungal phylotypes in the neutral-soil and acidic-soil communities in the pH-manipulation experiment. Occurrence indicates the number of samples in which the phylotypes occurred, and zero values imply that the phylotypes were not detected in this experiment, but detected in the trap culture surveys.
(DOCX)

S2 Table Frequency of the occurrence of arbuscular mycorrhizal fungal phylotypes in the trap culture surveys. ^a^Total number of site indicates the number of site in which the phylotypes occurred, and zero values imply that the phylotypes were not detected in these surveys, but detected in the pH-manipulation experiment.
(DOCX)

S3 Table S-D-R simplex analysis in the pH-manipulation experiment. ^a^Percentage relative contributions of phylotype turnover, richness agreement, and nestedness to the distribution pattern of arbuscular mycorrhizal fungi along the pH gradient are indicated. The same data matrices for the NODF/WNODF analysis were used.
(DOCX)

S4 Table Forward selection procedure by means of Monte Carlo permutation test in canonical correspondence analysis for the significance of environmental factors in driving arbuscular mycorrhizal fungal communities in the trap culture surveys. The presence/absence data (*n* = 61) was employed to assess the significance (999 permutations).
(DOCX)

S5 Table Mantel test for correlations between arbuscular mycorrhizal fungal communities and environmental factors across the six sites in the field surveys. Morisita-Horn distance was calculated in every combination of two out of the six sites as a measure of community dissimilarity using the phylotype-abundance data (9999 permutations).
(DOCX)

S6 Table S-D-R simplex analysis in the trap culture surveys. ^a^Percentage relative contributions of phylotype turnover, richness agreement, and nestedness to the distribution pattern of arbuscular mycorrhizal fungi along the pH gradient are indicated. The same data matrices for the NODF/WNODF analysis were used.
(DOCX)

S1 Text Surrounding vegetation and geographic features of Mukawa site. (DOCX)
