# Supplementary figures and images for: Nestedness in Arbuscular Mycorrhizal Fungal Communities along Soil pH Gradients in Early Primary Succession: Acid-Tolerant Fungi Are pH Generalists
Source: PLoS One. 2016 Oct 18;11(10):e0165035. doi: 10.1371/journal.pone.0165035 (PMC5068792; doi:10.1371/journal.pone.0165035)

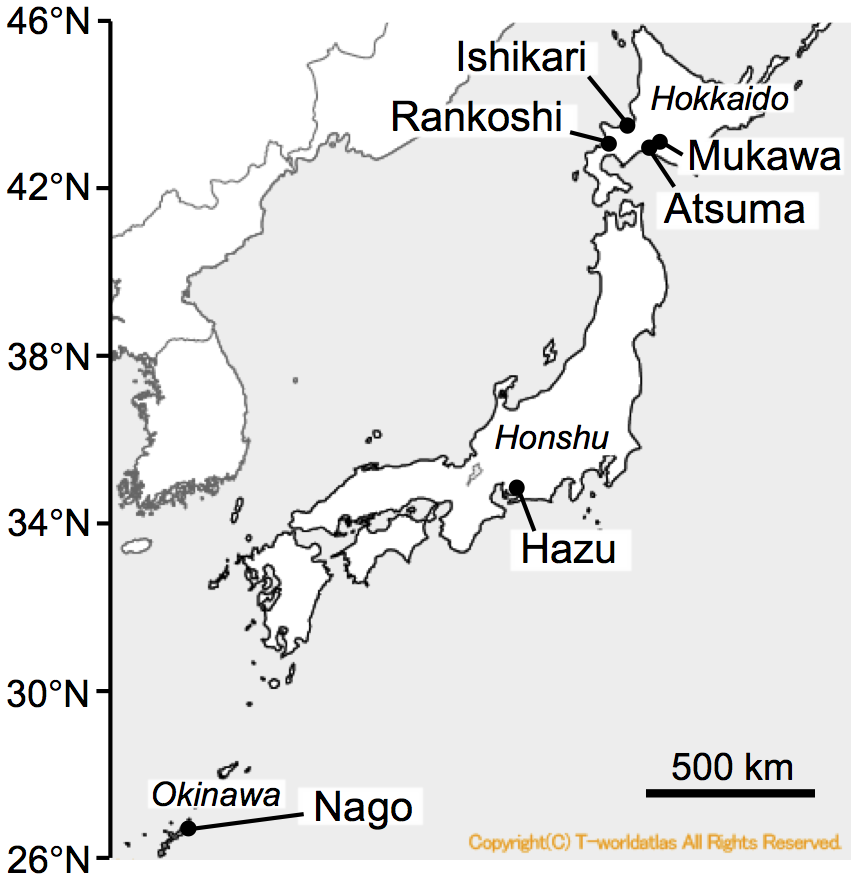

Supplement: S1 Fig — Soil types are acid sulfate soil in Rankoshi, Hazu, and Naog sites, dune soil in Ishikari, sandy loam soil in Atsuma, and ultramafic soil in Mukawa. Geographic/climatic data, vegetation, and soil chemical properties are summarized in Table 1. Reprinted from Sekai Chizu (http://www.sekaichizu.jp/) with permission from Itsuki Shoji Co., Ltd., original copyright 2009. (TIFF) [file pone.0165035.s001.tiff]

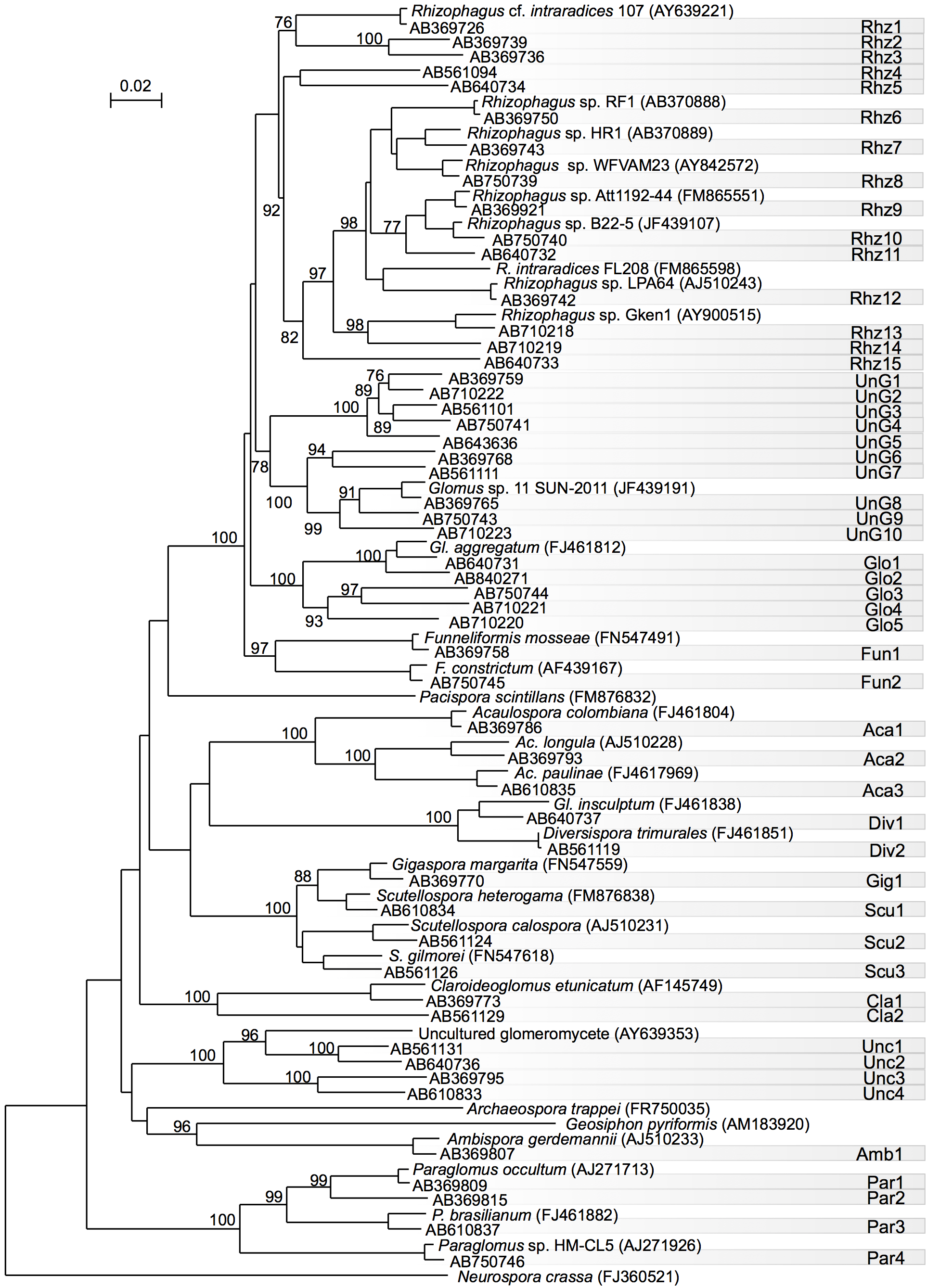

Supplement: S2 Fig — Representative sequences of large subunit ribosomal RNA gene of arbuscular mycorrhizal (AM) fungi obtained in the pH-manipulation experiment and trap culture surveys were aligned together with published sequences using Clustal X, and the tree was drawn by NJplot. Grey boxes represent AM fungal phylotypes that were defined based on ≥ 95% sequence similarities. Bootstrap values more than 70% are indicated. Genbank accession numbers of the reference and representative sequences are indicated. (TIFF) [file pone.0165035.s002.tiff]

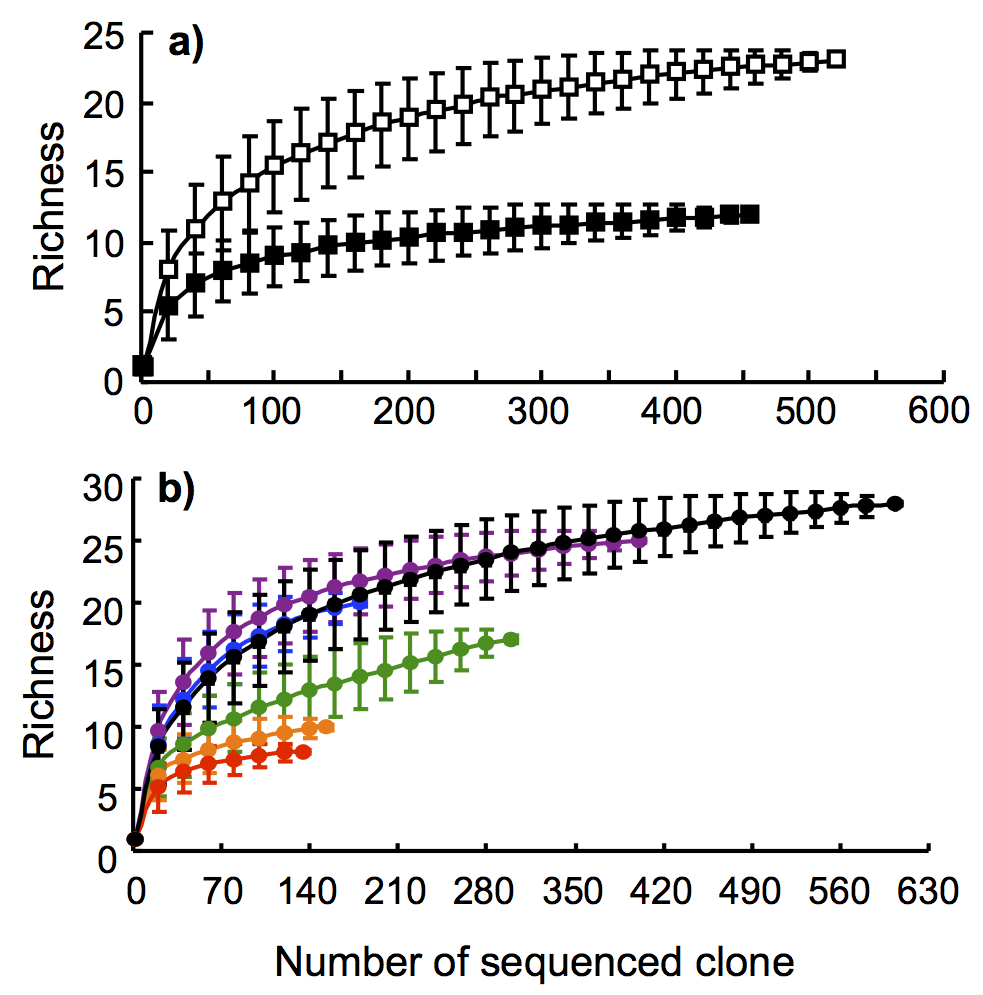

Supplement: S3 Fig — a) Trap culture surveys. Rhizosphere soils of M. sinensis were collected from Rankoshi (red), Hazu (yellow), Nago (green), Atsuma (blue), Ishikari (purple), and Mukawa (black) sites and subjected to trap culture with M. sinensis seedlings for community analysis. b) pH-manipulation experiment. Rhizosphere soils of M. sinensis were collected from Ishikari (open squares) and Rankoshi (closed squares) sites, designated as acidic soil- and neutral soil-AM fungal inocula, respectively, and subjected to trap culture with M. sinensis seedlings at three different pH. Total richness in the combined communities of all pH treatments was analyzed. The curves were constructed with Analytic Rarefaction 1.3. Bars indicate 95% CI. (TIFF) [file pone.0165035.s003.tiff]

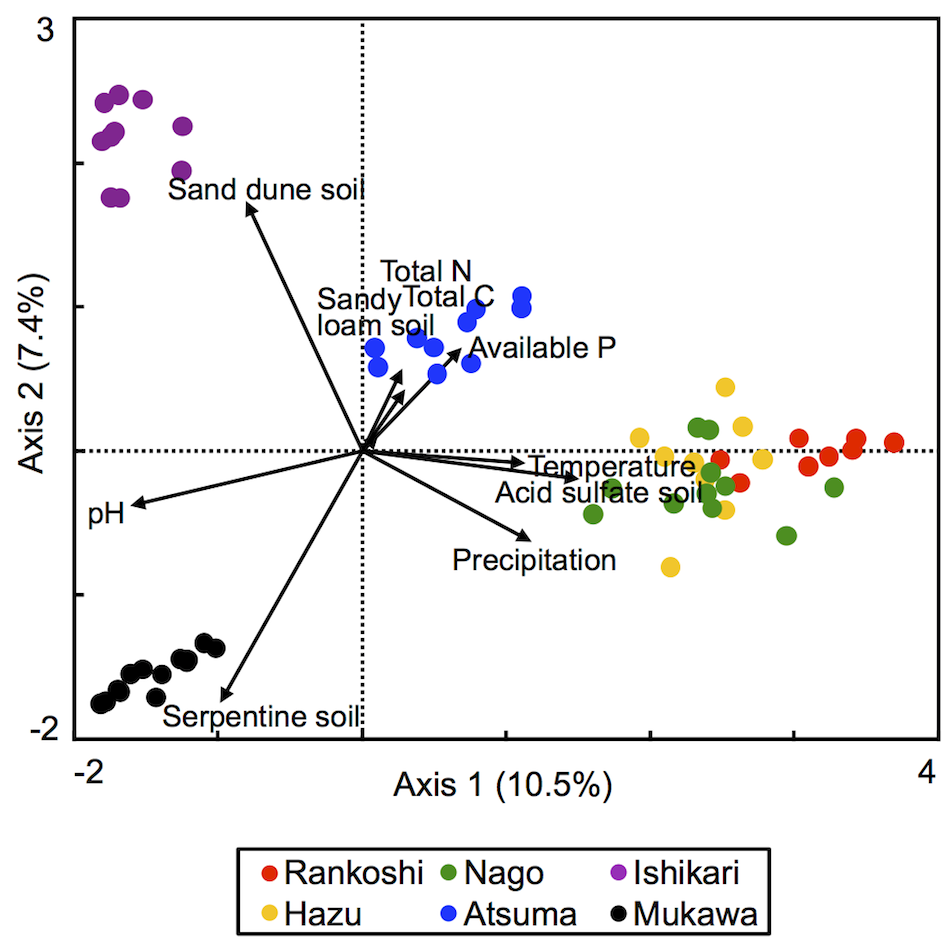

Supplement: S4 Fig — Rhizosphere soils of M. sinensis were collected from the six sites and subjected to soil trap culture with M. sinensis seedlings for community analysis. All the environmental variables used in this analysis are indicated as arrows, and the significance of the factors was assessed by forward selection procedure by means of Monte Carlo permutation test (S4 Table). (TIFF) [file pone.0165035.s004.tiff]
