# Supplementary material for: Nestedness in Arbuscular Mycorrhizal Fungal Communities along Soil pH Gradients in Early Primary Succession: Acid-Tolerant Fungi Are pH Generalists
Source: PLoS One. 2016 Oct 18;11(10):e0165035. doi: 10.1371/journal.pone.0165035 (PMC5068792; doi:10.1371/journal.pone.0165035)
Supplement: S1 Table — Occurrence indicates the number of samples in which the phylotypes occurred, and zero values imply that the phylotypes were not detected in this experiment, but detected in the trap culture surveys. (DOCX) [file pone.0165035.s005.docx]

**S1 Table. Frequency of the occurrence of AM fungal phylotypes in the neutral-soil and acidic-soil communities in the pH-manipulation experiment.**

|  | Neutral soil-community | | |  | Acidic soil-community | | |  |
| --- | --- | --- | --- | --- | --- | --- | --- | --- |
| Phylotype | pH 3.4 | pH 4.0 | pH 5.5 |  | pH 3.4 | pH 4.0 | pH 5.5 | Occurrence^a^ |
| Rhz1 | - | 2 | 3 |  | - | 1 | - | 3 |
| Rhz2 | 2 | 1 | 1 |  | 2 | 1 | 4 | 6 |
| Rhz3 | 1 | 2 | 1 |  | 2 | 1 | 1 | 6 |
| Rhz4 | - | - | - |  | - | - | - | 0 |
| Rhz5 | - | - | 1 |  | - | - | - | 0 |
| Rhz6 | 5 | 1 | 2 |  | 1 | 1 | 2 | 6 |
| Rhz7 | 4 | - | 4 |  | 1 | - | 1 | 4 |
| Rhz8 | - | - | 1 |  | - | - | - | 1 |
| Rhz9 | 1 | - | 1 |  | - | - | - | 2 |
| Rhz10 | - | - | - |  | - | - | - | 0 |
| Rhz11 | - | - | - |  | - | - | - | 0 |
| Rhz12 | 1 | - | 1 |  | 1 | - | - | 3 |
| Rhz13 | - | - | - |  | - | - | - | 0 |
| Rhz14 | - | - | - |  | - | - | - | 0 |
| Rhz15 | - | - | - |  | - | - | - | 0 |
| UnG1 | 3 | 3 | 3 |  | 2 | - | 1 | 5 |
| UnG2 | - | - | - |  | - | - | - | 0 |
| UnG3 | 3 | 1 | 1 |  | - | - | - | 3 |
| UnG4 | - | - | - |  | - | - | - | 0 |
| UnG5 | - | - | - |  | - | - | - | 0 |
| UnG6 | - | - | - |  | - | - | - | 0 |
| UnG7 | - | - | - |  | - | - | - | 0 |
| UnG8 | - | 1 | 1 |  | - | - | - | 2 |
| UnG9 | - | - | - |  | - | - | - | 0 |
| UnG10 | - | - | 1 |  | - | - | - | 1 |
| Glo1 | - | - | - |  | - | - | - | 0 |
| Glo2 | - | - | - |  | - | - | - | 0 |
| Glo3 | - | - | - |  | - | - | - | 0 |
| Glo4 | - | - | - |  | - | - | - | 0 |
| Glo5 | - | - | - |  | - | - | - | 0 |
| Fun1 | - | - | 1 |  | - | - | - | 1 |
| Fun2 | - | - | - |  | - | - | - | 0 |
| Aca1 | 2 | 2 | 2 |  | - | - | - | 3 |
| Div2 | - | - | - |  | - | - | - | 0 |
| Gig1 | - | - | - |  | - | - | - | 0 |

(continues to the next sheet)

**S1 Table.** (continued)

|  | Neutral soil-community | | |  | Acidic soil-community | | |  |
| --- | --- | --- | --- | --- | --- | --- | --- | --- |
| Phylotype | pH 3.4 | pH 4.0 | pH 5.5 |  | pH 3.4 | pH 4.0 | pH 5.5 | Occurrence^a^ |
| Scu1 | 3 | 2 | 1 |  | 1 | - | - | 4 |
| Scu2 | - | 1 | 2 |  | 5 | 1 | 1 | 5 |
| Scu3 | - | - | 1 |  | - | - | - | 1 |
| Cla1 | - | - | - |  | - | - | - | 0 |
| Cla2 | - | - | - |  | - | - | - | 0 |
| Unc1 | - | - | - |  | - | - | - | 0 |
| Unc2 | 1 | - | - |  | - | - | - | 1 |
| Unc3 | - | - | - |  | - | - | - | 0 |
| Unc4 | - | - | - |  | - | - | - | 0 |
| Amb1 | - | - | 2 |  | - | - | - | 1 |
| Par1 | 1 | - | 3 |  | 2 | 2 | 4 | 5 |
| Par2 | 2 | 2 | 5 |  | 5 | 5 | 5 | 6 |
| Par3 | - | - | - |  | - | - | - | 0 |
| Par4 | - | - | - |  | - | - | - | 0 |
| Clone no. | 190 | 114 | 216 |  | 159 | 104 | 192 |  |
| Richness | **14** | **12** | **22** |  | **10** | **7** | **9** |  |

^a^ Occurrence indicates the number of samples in which the phylotypes occurred, and zero values imply that the phylotypes were not detected in this experiment, but detected in the trap culture surveys.
