# Supplementary material for: Nestedness in Arbuscular Mycorrhizal Fungal Communities along Soil pH Gradients in Early Primary Succession: Acid-Tolerant Fungi Are pH Generalists
Source: PLoS One. 2016 Oct 18;11(10):e0165035. doi: 10.1371/journal.pone.0165035 (PMC5068792; doi:10.1371/journal.pone.0165035)
Supplement: S2 Table — aTotal number of site indicates the number of site in which the phylotypes occurred, and zero values imply that the phylotypes were not detected in these surveys, but detected in the pH-manipulation experiment. (DOCX) [file pone.0165035.s006.docx]

**S2 Table. Frequency of the occurrence of AM fungal phylotypes in the trap culture surveys.**

|  | Site (collection year) | | | | | | | |  |
| --- | --- | --- | --- | --- | --- | --- | --- | --- | --- |
|  | Rankoshi | Hazu | Nago | | Atsuma | Ishikari | | Mukawa | Total no. of site^a^ |
| Phylotype | (2005) | (2005) | (2006) | (2007) | (2006) | (2007) | (2008) | (2009) |  |
| Rhz1 | 6 | 7 | - | 1 | 3 | - | - | 1 | 5 |
| Rhz2 | 3 | - | - | - | 1 | - | - | - | 2 |
| Rhz3 | 2 | - | - | - | - | - | - | - | 1 |
| Rhz4 | - | - | - | - | - | 1 | 1 | - | 1 |
| Rhz5 | - | - | - | - | - | 1 | - | - | 1 |
| Rhz6 | - | 3 | 6 | 2 | 4 | - | - | 2 | 4 |
| Rhz7 | - | 2 | - | - | 5 | 1 | - | 7 | 4 |
| Rhz8 | - | - | - | - | - | - | - | - | 0 |
| Rhz9 | - | 1 | 1 | - | 3 | 1 | 1 | 7 | 5 |
| Rhz10 | - | - | - | - | - | - | - | 6 | 1 |
| Rhz11 | - | - | - | - | - | 1 | - | 5 | 2 |
| Rhz12 | - | - | 2 | - | - | - | - | - | 1 |
| Rhz13 | - | - | 1 | - | - | - | - | - | 1 |
| Rhz14 | - | - | - | 1 | - | - | - | - | 1 |
| Rhz15 | - | - | - | - | - | - | 1 | - | 1 |
| UnG1 | 1 | 3 | 2 | 1 | 3 | - | - | 2 | 5 |
| UnG2 | 1 | - | - | 1 | - | - | - | - | 2 |
| UnG3 | - | - | - | - | - | 2 | 2 | 9 | 2 |
| UnG4 | - | - | - | - | - | - | - | 5 | 1 |
| UnG5 | - | - | - | - | - | - | 1 | - | 1 |
| UnG6 | - | - | - | - | 4 | 2 | 2 | 1 | 3 |
| UnG7 | - | - | - | - | - | - | 3 | - | 1 |
| UnG8 | 1 | - | - | - | 3 | 3 | 1 | 3 | 4 |
| UnG9 | - | - | - | - | - | - | - | 3 | 1 |
| UnG10 | - | - | - | - | - | - | - | - | 0 |
| Glo1 | - | - | - | - | - | - | 1 | 1 | 2 |
| Glo2 | - | - | - | - | - | - | - | 2 | 1 |
| Glo3 | - | - | - | - | - | - | - | 2 | 1 |
| Glo4 | - | - | - | - | 1 | - | - | 2 | 2 |
| Glo5 | - | - | - | - | 1 | - | - | 2 | 2 |
| Fun1 | - | - | - | - | 1 | - | - | - | 1 |
| Fun2 | - | - | - | - | - | - | - | 2 | 1 |

(continues to the next sheet)

**S2 Table.** (continued)

|  | Site (collection year) | | | | | | | |  |
| --- | --- | --- | --- | --- | --- | --- | --- | --- | --- |
|  | Rankoshi | Hazu | Nago | | Atsuma | Ishikari | | Mukawa | Total no. of site^a^ |
| Phylotype | (2005) | (2005) | (2006) | (2007) | (2006) | (2007) | (2008) | (2009) |  |
| Aca1 | - | 9 | 4 | 1 | 2 | 1 | - | 1 | 5 |
| Aca2 | - | - | 1 | - | 5 | 1 | 1 | - | 3 |
| Aca3 | - | - | - | - | 1 | 2 | 2 | - | 2 |
| Div1 | - | - | - | - | - | - | 3 | 2 | 2 |
| Div2 | - | - | - | - | - | 1 | - | 5 | 2 |
| Gig1 | - | 1 | 1 | - | 2 | - | - | 1 | 4 |
| Suc1 | - | - | 1 | 1 | 1 | - | - | 1 | 3 |
| Suc2 | - | - | - | - | - | 1 | 4 | - | 1 |
| Suc3 | - | - | - | - | - | - | 1 | - | 1 |
| Cla1 | - | - | 1 | - | - | - | 2 | 8 | 3 |
| Cla2 | - | - | - | - | - | - | 2 | - | 1 |
| Unc1 | - | - | - | - | - | 2 | 3 | 12 | 2 |
| Unc2 | - | - | - | - | - | 2 | 1 | - | 1 |
| Unc3 | - | 1 | - | 1 | - | - | - | - | 2 |
| Unc4 | - | - | - | 1 | - | - | - | 2 | 2 |
| Amb1 | 1 | 1 | 8 | 1 | 3 | - | - | - | 4 |
| Par1 | 2 | 4 | 2 | 1 | 1 | - | - | 1 | 5 |
| Par2 | - | - | - | - | 7 | 3 | 3 | - | 2 |
| Par3 | - | - | - | - | 1 | 2 | 5 | - | 2 |
| Par4 | - | - | - | - | - | - | - | 10 | 1 |
| Sample no. |  |  | 9 | 2 |  | 4 | 6 |  |  |
|  | 8 | 9 | 11 | | 10 | 10 | | 13 |  |
| Clone no. |  |  | 112 | 182 |  | 232 | 158 |  |  |
|  | 134 | 153 | 294 | | 176 | 390 | | 604 |  |
| Richness | **8** | **10** | **17** | | **20** | **25** | | **28** |  |

^a^ Total number of site indicates the number of site in which the phylotypes occurred, and zero values imply that the phylotypes were not detected in these surveys, but detected in the pH-manipulation experiment.
