# Supplementary material for: Nestedness in Arbuscular Mycorrhizal Fungal Communities along Soil pH Gradients in Early Primary Succession: Acid-Tolerant Fungi Are pH Generalists
Source: PLoS One. 2016 Oct 18;11(10):e0165035. doi: 10.1371/journal.pone.0165035 (PMC5068792; doi:10.1371/journal.pone.0165035)
Supplement: S3 Table — aPercentage relative contributions of phylotype turnover, richness agreement, and nestedness to the distribution pattern of arbuscular mycorrhizal fungi along the pH gradient are indicated. The same data matrices for the NODF/WNODF analysis were used. (DOCX) [file pone.0165035.s007.docx]

**S3 Table.** **S-D-R simplex analysis in the pH-manipulation experiment.**

| Data format | Turnover | Richness agreement | Nestedness |
| --- | --- | --- | --- |
| Presence/absence | 49.5^a^ | 66.2 | 84.3 |
| Abundance | 65.0 | 69.7 | 65.4 |

^a^ Percentage relative contributions of phylotype turnover, richness agreement, and nestedness to the distribution pattern of AM fungi along the pH gradient are indicated. The same data matrices for the NODF/WNODF analysis were used.
