# Supplementary material for: Nestedness in Arbuscular Mycorrhizal Fungal Communities along Soil pH Gradients in Early Primary Succession: Acid-Tolerant Fungi Are pH Generalists
Source: PLoS One. 2016 Oct 18;11(10):e0165035. doi: 10.1371/journal.pone.0165035 (PMC5068792; doi:10.1371/journal.pone.0165035)
Supplement: S4 Table — The presence/absence data (n = 61) was employed to assess the significance (999 permutations). (DOCX) [file pone.0165035.s008.docx]

**S4 Table. Forward selection procedure by means of Monte Carlo permutation test in canonical correspondence analysis for the significance of environmental factors in driving arbuscular mycorrhizal fungal communities in the trap culture surveys.**

| Factor | Pseud-*F* | *P* |
| --- | --- | --- |
| pH | 6.21 | 0.002 |
| Serpentine soil | 4.56 | 0.002 |
| Temperature | 2.96 | 0.002 |
| Sandy loam soil | 2.57 | 0.002 |
| Precipitation | 2.03 | 0.004 |
| Acid sulfate soil | 1.53 | 0.028 |
| Total N | 1.30 | 0.140 |
| Total C | 1.09 | 0.338 |
| Available phosphate | 1.03 | 0.364 |

The presence/absence data (*n* = 61) was employed to assess the significance (999 permutations).
