# Supplementary material for: Nestedness in Arbuscular Mycorrhizal Fungal Communities along Soil pH Gradients in Early Primary Succession: Acid-Tolerant Fungi Are pH Generalists
Source: PLoS One. 2016 Oct 18;11(10):e0165035. doi: 10.1371/journal.pone.0165035 (PMC5068792; doi:10.1371/journal.pone.0165035)
Supplement: S5 Table — Morisita-Horn distance was calculated in every combination of two out of the six sites as a measure of community dissimilarity using the phylotype-abundance data (9999 permutations). (DOCX) [file pone.0165035.s009.docx]

**S5 Table. Mantel test for correlations between arbuscular mycorrhizal fungal communities and environmental factors across the six sites in the field surveys.**

| Factor | Control for | *r* | *P* |
| --- | --- | --- | --- |
| pH | - | 0.755 | 0.010 |
| Total N | - | -0.494 | 0.973 |
| Total C | - | -0.471 | 0.937 |
| Available P | - | -0.362 | 0.846 |
| Geographic distance | - | -0.051 | 0.511 |
| Latitude | - | -0.040 | 0.508 |
| Precipitation | - | 0.101 | 0.391 |
| Temperature | - | -0.085 | 0.590 |
| Total N | pH | -0.526 | 0.977 |
| Total C | pH | -0.530 | 0.973 |
| Available P | pH | -0.550 | 0.980 |
| Geographic distance | pH | 0.018 | 0.466 |
| Latitude | pH | 0.046 | 0.408 |
| Precipitation | pH | 0.269 | 0.237 |
| Temperature | pH | -0.008 | 0.464 |

Morisita-Horn distance was calculated in every combination of two out of the six sites as a measure of community dissimilarity using the phylotype-abundance data (9999 permutations).
