# Supplementary material for: Nestedness in Arbuscular Mycorrhizal Fungal Communities along Soil pH Gradients in Early Primary Succession: Acid-Tolerant Fungi Are pH Generalists
Source: PLoS One. 2016 Oct 18;11(10):e0165035. doi: 10.1371/journal.pone.0165035 (PMC5068792; doi:10.1371/journal.pone.0165035)
Supplement: S1 Text — (DOCX) [file pone.0165035.s011.docx]

**S1 Text. Surrounding vegetation and geographic features of Mukawa site.**

Mukawa site is an old serpentine quarry abandoned in the 1990s (400 in length × 200 m in width) on a shallow slope (10–13˚) in Mt. Bozu (790 m altitude). The area is surrounded by secondary forest of *Betula platyphylla* (Betulaceae), *Quercus* spp. (Fagaceae), *Acer* spp. (Aceraceae) as well as *Larix kaempferi* (Pinaceae) plantation. Although quarrying had ceased several years ago, development of vegetation is still poor due to generation of ultramafic soil. *M. sinensis* is distributed only patchily, but the most dominant species in the site. The herbs *Veronica schmidtiana* Regal var. *yezoalpina* Yamazaki and *Anaphalis lctea* Maxim were also observed, but occur much less frequently than *M. sinensis*.
